# Aberrant DR5 transport through disruption of lysosomal function suggests a novel mechanism for receptor activation

**DOI:** 10.18632/oncotarget.11073

**Published:** 2016-08-05

**Authors:** Birce Akpinar, Barbora Safarikova, Jarmila Laukova, Shubhranshu Debnath, Alena Hyrslova Vaculova, Boris Zhivotovsky, Magnus Olsson

**Affiliations:** ^1^ Division of Toxicology, Institute of Environmental Medicine, Karolinska Institutet, Stockholm, Sweden; ^2^ Department of Cytokinetics, Institute of Biophysics, Academy of Sciences of the Czech Republic, v.v.i., Brno, Czech Republic

**Keywords:** apoptosis, autophagy, chloroquine, DR5, lysosomes

## Abstract

To examine reciprocal or unilateral implications between two cell destruction processes, autophagy and apoptosis, in 5-Fluorouracil (5-FU)-treated tumor cells, a combination of chemical inhibitors, RNAi and genetic approaches were used. In contrast to cancer cells harboring obstructed apoptosis, either at the DISC or the mitochondrial level, p53-deficiency generated signs of autophagy deregulation upon chemotherapy. On the other, hand disruption of lysosomal function by chloroquine, caused a profound decrease in apoptotic markers appearing in response to 5-FU. DR5, which is essential for 5-FU-induced apoptosis, accumulated in lysosomes and autophagosomes upon chloroquine treatment. Since neither 3-MA, RNAi of critical autophagy regulators or inhibition of cathepsins reversed apoptosis in a similar manner, it is likely that not autophagy *per se* but rather correct receptor transport is an important factor for 5-FU cytotoxicity. We found that apoptosis generated by TRAIL, the cognate ligand for DR5, remained unchanged upon chloroquine lysosomal interference, indicating that 5-FU activates the receptor by a discrete mechanism. In support, depletion of membrane cholesterol or hampering cholesterol transport drastically reduced 5-FU cytotoxicity. We conclude that targeting of lysosomes by chloroquine deregulates DR5 trafficking and abrogates 5-FU- but not TRAIL-stimulated cell elimination, hence suggesting a novel mechanism for receptor activation.

## INTRODUCTION

The tumor necrosis factor receptor (TNFR) superfamily regulates a variety of cellular processes, including development of peripheral lymphoid organ and termination of undesirable cells during immune responses. TNFRs are activated by ligands of the tumor necrosis factor (TNF) family, most of which are type II membrane protein trimers that may become soluble as a consequence of proteolytic cleavage. Active TNFR family members oligomerize and intracellular signaling occurs as a result of cytoplasmic factor recruitments. Death receptors 4 and 5 (DR4, DR5; TRAILR1, TRAILR2) are stimulated by the TNF-related apoptosis-inducing ligand (TRAIL, Apo2L), which unlike other TNF family members preferentially induces cell death in tumor cells [1, 2], suggesting a prospective therapeutic application in cancer treatment. Once activated, DR homotypic domain interactions promote death inducing signaling complex (DISC) formation, which allows caspase-8 and −10 processing. This is followed by engagement of the apoptotic cascade, including mitochondrial destabilization by means of the BH3-Interacting Domain Death Agonist (Bid)-Bcl-2-Associated X Protein (BAX)/Bcl-2-Antagonist/Killer 1 (BAK) axis and effector caspase activation. However, several lines of evidence suggest that DRs are more dynamic than previously anticipated, both with respect to function and cellular localization. For example, endocytotic internalization and ensuing lysosomal localization of active DR5 was suggested to promote apoptosis in malignant liver cell lines by means of endopeptidase release [3]. In support, it was described that TRAIL induces recruitment of the phosphofurin acidic cluster sorting protein-2 (PACS-2) to DR5-positive endosomes allowing for complex formation with Bim and Bax on lysosomal membranes [4]. Accumulation of intracellular DR5 accumulation was reported to occur as a response to persistent ER stress, resulting in ligand-independent receptor activation and apoptosis through caspase-8 activity [5]. Overexpression of DR4 and DR5 has been demonstrated in several cancers, although not in the expected plasma membrane location, but rather in cytoplasmic and nuclear compartments [6]. In addition, correlation of the expression status with clinical parameters showed prevalent prognostic significance of high receptor content, identifying predominantly DR5 as a negative predictive marker for disease outcome. Consequently, apart from apoptosis and in some cases necroptosis [7], DR5 may potentiate signaling for cell survival [8], which has been confirmed in functional assays. For instance, increased metastasis of pancreatic tumors was observed following TRAIL treatment and nuclear DR5 has been implicated in suppression of miRNA let-7 maturation, which leads to proliferation of pancreatic cancer cell lines [9, 10]. Apart from being stimulated by TRAIL harbored on or released from immune cells, recent data indicate that diverse tumor treatment strategies enable the activation of DRs in an autocrine or paracrine manner. In acute myeloid leukemia (AML) cells, tumor selectivity of HDAC inhibitors involves expression of TRAIL and subsequent activation of DR5 [11]. Moreover, retinoids and interferons have been demonstrated to stimulate cell death by means of TRAIL [12]. Similarly, our recent work emphasized that 5-Fluorouracil (5-FU) can induce apoptosis following the activation of DRs via an intracellular TRAIL-stimulated mechanism [13]. In the present study, initiated with the aim to analyze crosstalk between apoptosis and autophagy, we demonstrate that cell death generated by 5-FU requires cytoplasmic transport of DR5, which in turn seems to be tightly associated with free cholesterol. Hence, our data contributes to DR biology by indicating a novel activation mechanism for DR5.

## RESULTS

### In contrast to the obstruction of 5-FU-induced apoptosis at the DISC or mitochondrial level, p53 deficiency deregulates treatment-provoked autophagy

**Figure 1:**
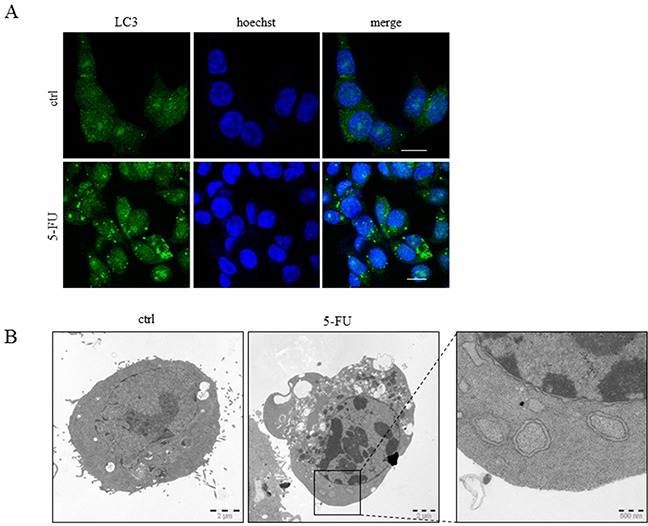
Autophagy is induced by 5-FU in HCT116 cells HCT116 cells were treated with 5-FU (768 μM) for 24 h and analysis of autophagy was accomplished by immunofluorescence detection of LC3 punctuation using a specific antibody. Bars, 10 μm **A.** Using the same experimental conditions as in (A) representative transmission electron microscope images of sections were prepared from control and 5-FU-treated cells including magnification of a region indicating membrane bi-layered autophagosomes **B.**

**Figure 2:**
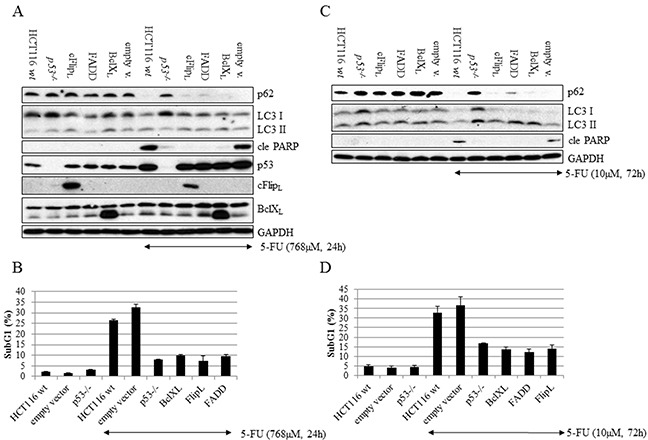
In contrast to disruption of autophagy by p53-deficiency, interference with apoptosis either at the DISC or the mitochondrial level did not influence the autophagy process c-FLIP_L_, FADD-DN or Bcl-X_L_ cDNA's were stably introduced into HCT116 *wt* cells by retroviral transduction and analyzed by western blotting and flow cytometry along with HCT116 *p53^−/−^* cells and relevant controls. Non-transduced, empty vector-, FADD-DN-, c-FLIP_L_- and Bcl-X_L_-containing as well as HCT116 *p53^−/−^* cells were harvested at the indicated time after 5-FU treatment and immuno-detection of cleaved PARP served as a marker of apoptosis, whereas p62 and LC3 staining were used as indicators of autophagy. Expression analyses of cFLIP_L_, FADD-DN and Bcl-X_L_ were accomplished using specific antibodies. Probing of GAPDH was used to confirm equal loading of the samples. Cells were treated with 5-FU, either using 768 μM for 24 h **A.** or 10 μM for 72 h **C.** In parallel and using identical experimental conditions, general cell death was examined by propidium iodide labeling of fixed cells followed by flow cytometry analysis of SubG1 populations **B.** and **D.**

Chemotherapy-provoked autophagy in tumor cells may either promote survival or death [14]. Consequently, therapeutic autophagy interference as an approach to enhance the effects of antineoplastic agents raises essential questions concerning the mechanistic interactions between this process and others, which are equally or more important for treatment efficacy, including apoptosis, necroptosis and senescence. Stimulation of autophagy by 5-FU has previously been demonstrated in non-small cell lung cancer A549 cells [15], and was confirmed in the colon carcinoma HCT116 cell line by the detection of LC3 punctuation in ICC analysis, the formation of autophagosomes by transmission electron microscopy (TEM) and p62 degradation as well as LC3-I to II conversion in SDS-PAGE (Figure 1A and 1B, Figure 2A and 2C). In order to examine cross-talk between apoptosis and autophagy in 5-FU-treated colon cancer cells, apoptosis ability was reduced by the stable overexpression of inhibitory factors. The cellular FADD-like IL-1β-converting enzyme-Inhibitory Protein (cFLIP_L_) and the FAS-Associated protein with Death Domain-Dominant Negative (FADD-DN) were used to reduce DISC formation, whereas B-cell lymphoma-extra-large (Bcl-X_L_) overexpression compromised cell death signaling at the mitochondrial level. In addition, a p53-deficient HCT116 cell line, where apoptosis is antagonized in a broad-spectrum manner, was included in the experiment. While all of these genetic alterations diminished the appearance of a subG1 population and the generation of cleaved Poly(ADP-ribose)polymerase (PARP) after 24 hours of 5-FU treatment (Figure 2A and 2B), only p53-deficiency had a pronounced effect on autophagy progress, as verified by a lack of p62 degradation (Figure 2A). The experiment was also performed using a clinically relevant dose of 5-FU (10 μM) for 72 hours where the data patterns with respect to markers of apoptosis and p62 degradation were verified (Figure 2C and 2D). Here, an overall increase in LC3-II was observed in treated FADD-DN, Bcl-X_L_ and cFlip_L_ overexpressing cells, most likely a consequence of the fact that these populations, in contrast to treated controls, have a higher fraction of surviving cells. As an enhanced LC3 protein level was observed in p53-deficient HCT116 cells, a comparison of autophagy progress between *wt* and *p53^−/−^*cells with respect to this particular marker could not be accomplished (Figure 2A and 2C). However, in respect to p62 a distinct degradation of this autophagy marker could be detected in p53 *wt* but not in *p53^−/−^.* cells. Thus, in contrast to the obstruction of p53 function, specific hindrance of apoptosis either at the DISC or mitochondrial level did not interfere with 5-FU-induced autophagy in HCT116 cells. Accordingly, it appears that regulation of autophagy by p53 mainly depends on mechanisms other than apoptosis [16].

### In contrast to inhibition of autophagy by several strategies, chloroquine efficiently reduces 5-FU-generated apoptosis

**Figure 3:**
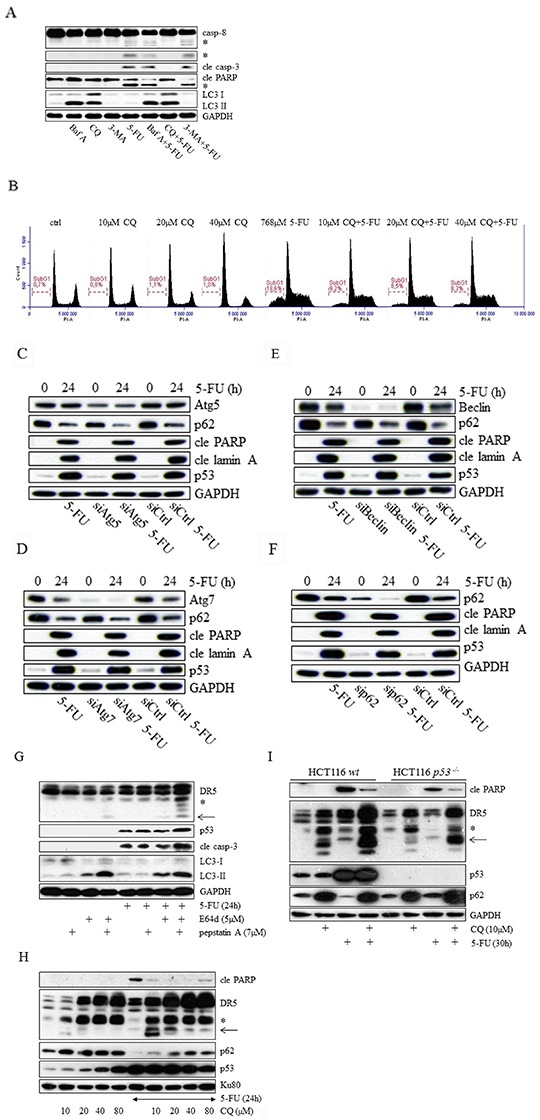
Chloroquine and bafilomycin A abrogate 5-FU generated apoptosis in HCT116 cells through an effect separated from autophagy inhibition The effect of 5-FU with respect to the processing of caspases −8, −3 and PARP was analyzed by immunoblotting using total protein lysates from cells in which autophagy had been compromised by means of chloroquine (CQ, 20 μM), bafilomycin A (Baf A, 100 nM) or 3-methyladenine (3-MA, 5 mM) treatments. Detection of LC3 was performed to verify drug activities **A.** Cytotoxicity of 5-FU (768 μM, 20h), either used as a single agent or in combination with 10, 20 or 40 μM chloroquine (CQ), was examined by propidium iodide cell labeling followed by FACS analysis of SubG1 populations **B.** Along with siControl cells, siAtg5 **C.** siAtg7 **D.** siBeclin **E.** and siSQSTM1 (p62) **F.** transfected (20 nM) HCT116 *wt* cells were either left untreated or treated with 768 μM 5-FU for 24 h. Western blot examination of cell lysates with respect to cleaved lamin A (cle lamin A), cleaved PARP (cle PARP), p53 and siRNA efficiency are shown in (C–F). The apoptotic effects of 5-FU (768 μM), either alone or in co-treatments using cathepsin inhibitors E64d (5 μM) or pepstatin A (7 μM), or their combination, were analyzed by SDS-PAGE. Immuno-detection of DR5, p53, cleaved caspase-3 (cle casp-3) and LC3 in SDS-PAGE is outlined. **G.** Expression of DR5 and p53 in HCT116 *wt* cells as a result of CQ (10, 20, 40 or 80 μM) or 5-FU (768 μM) treatment, or their combinations was investigated by SDS-PAGE using HCT116 *wt* protein isolates. Immuno-detection of p62 and cleaved PARP served as markers for autophagy and apoptosis, respectively **H.** Along with controls, HCT116 *wt* and p5*3^−/−^* cells were treated with 5-FU (768 μM) or CQ (10 μM), or their combination. Isolated protein lysates were then separated by SDS-PAGE in order to analyze DR5, cleaved PARP, p53 and p62 using specific antibodies **I.** Immuno-detection of GAPDH or KU80 was used to control for equal loading of samples in (A and C–I). Processed caspase-8 fragments and the short isoform of DR5 are indicated by asterisks (A and G–I). An additional DR5-related fragment detected in western blot and appearing in CQ treated as well as in E64d and pepstatin A co-treated samples is indicated by an arrow (G–I).

During autophagy, the formation of autophagosomes and autolysosomes is followed by the degradation of intra-autophagosomal material. To analyze crosstalk between autophagosomal turnover and 5-FU-stimulated apoptosis, we employed three frequently used chemical autophagy inhibitors, 3-methyladenine (3-MA), bafilomycin A1 (Baf A) and chloroquine (CQ). The first is an inhibitor of phosphatidylinositol 3-kinases (PI3K)-mediated mTOR activity and the other two interfere with lysosomal acidification. As determined by the activation of caspase-8, the most apical caspase in this experimental system [13], CQ reduced its processing substantially while Baf A was less potent and 3-MA interfered insignificantly (Figure 3A, Supplementary Figure S1A and S1B). However, although Baf A reduced 5-FU-induced caspase-8 activation, no apparent effect was detected on downstream apoptotic markers, such as the processing of caspase-3 or cleaved PARP (Figure 3A). Since CQ treatment showed the most prominent effect on the response to 5-FU-cytotoxicity in HCT116 cells, subsequent experiments were focused on this particular mechanism and data were verified using the RKO and HT29 cell lines (Supplementary Figures S1C and S1D). In agreement with the western blot results, a decrease in the 5-FU-generated subG1 population was observed using CQ concentrations ranging from 10 to 40 μM (Figure 3B). Staining of fixed cells with propidium iodide did not reveal any changes in the cell cycle distribution (Figure 3B), thus excluding cell cycle aberrations as a potential source for the cell death inhibitory effects. These data support a functional connection between apoptosis signaling and lysosome function rather than the involvement of autophagy, a model supported by RNAi silencing of the essential autophagy-related proteins such as Atg 5, Atg7, Beclin and p62 (Figure 3C–3F). In case of Atg7 and Beclin, identical results were independently achieved by two distinct siRNA's relating to each factor. Insignificance of Atg7 and Beclin for 5-FU-induced apoptosis was also verified in *p53^−/−^* HCT116 cells (data not shown). A plausible explanation for these observations would be that DR5 promotes lysosomal permeabilization and the sequential release of proteases, as suggested by Akazawa et al [3]. E64d, a membrane-permeable inhibitor of cathepsins B, H, and L, and pepstatin A, an inhibitor of cathepsins D and E were used to assess this possibility. However, as single agents neither of these compounds influenced chemotherapy efficiency, determined by the appearance of effector caspase-3 processing. In fact, their combination rather promoted 5-FU-toxicity, possibly through the enhancement of p53 stability (Figure 3G). By comparison, notwithstanding that CQ concentrations ranging from 20 to 80 μM elevate p53 and DR5 significantly, and that the levels of these proteins are further promoted by the addition of 5-FU, CQ efficiently reduces chemotherapy-toxicity, as verified by a decrease in PARP cleavage (Figure 3H). *DR5* has been identified as a p53 transcriptionally activated gene [17], but in contrast to the response to 5-FU alone, western blot analysis of treated p53-deficient HCT116 cells clearly showed that CQ either stimulates DR5 protein expression or inhibits receptor degradation by mechanisms distinct from p53. Nevertheless, apparent inhibition of 5-FU cytotoxicity by CQ was also shown in cells lacking p53, (Figure 3I). Thus, disruption of lysosomal activity by CQ efficiently abrogates 5-FU-induced apoptosis in the colon carcinoma cell line investigated. This mechanism does not involve autophagy, or the activities of p53 or cathepsins.

### CQ and Baf A but not 3-MA treatments are associated with lysosomal and autophagosomal localization of DR5

**Figure 4:**
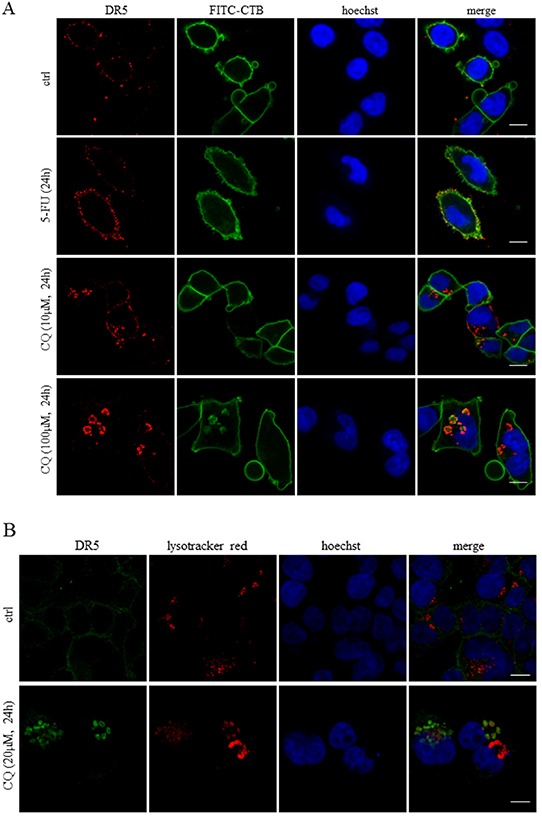

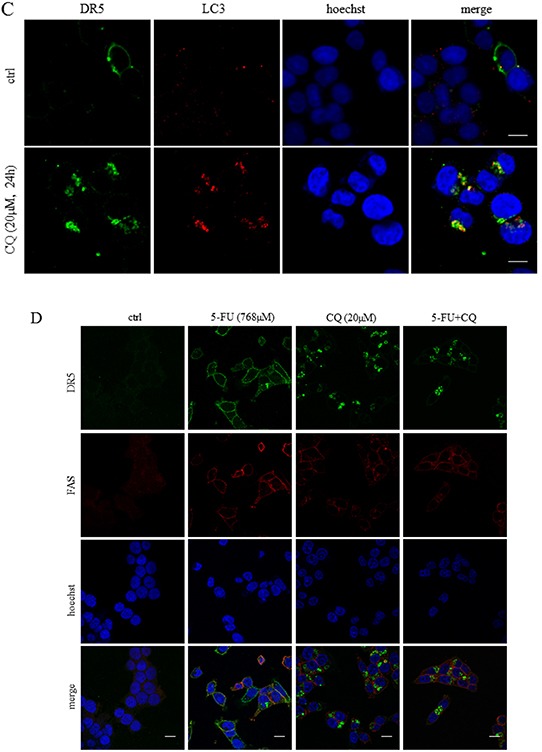
Chloroquine treatment leads to cytosolic DR5 accumulation overlapping with lysosomes and autophagosomes Along with control cells, HCT116 *wt* cells treated with 5-FU (768 μM) or CQ (10 or 100 μM) for 24h were fixed in 3.8 % formaldehyde and stained with a DR5-specific antibody (11B4) (red). The plasma membranes were counterstained by FITC-conjugated CTB (green) **A.** Similarly, cells were treated as indicated and stained for DR5 (green) and lysosomes (red) **B.** as well as DR5 (green), LC3 (red). **C.** Co-localizations were investigated by means of the lysotracker red dye and specific antibodies, respectively. Cellular localizations of the FAS (red) and DR5 (green) receptors in control, 5-FU (768 μM), CQ (20 μM) or combinatorial treatment are outlined in **D.** All samples were analyzed by confocal microscopy. Cell nuclei were counterstained by the Hoechst 33342 stain (blue). Bars, 10 μM.

Since DR5 is one of the most apical apoptotic factors in the signaling cascade initiated by 5-FU in the current experimental system, a thorough investigation of receptor localization in control and treated cells was performed. Treatment with 5-FU generated an apparent plasma membrane accumulation of DR5, as verified by co-localization with FITC-conjugated cholera toxin B (FITC-CTB). On the other hand, 10 μM CQ delocalized the receptor into a cytoplasmic punctate pattern, which became membrane-enclosed ring forming structures at a concentration of 100 μM (Figure 4A). Notably, cytoplasmic DR5 structures formed in consequence to CQ treatment co-localized partly with both lysotracker red staining and the autophagosomal marker LC3 (Figures 4B and 4C). Treatment with Baf A but not 3-MA induced a DR5 cytoplasmic localization pattern analogous to that of CQ-treated cells (Supplementary Figures S2A and S2B). Differently to DR5, the death receptor FAS (APO-1, CD95) appeared exclusively in the plasma membrane in response to both CQ and 5-FU alone, or in combination (Figure 4D). Yet, as both transferrin and DR4 form comparable cytoplasmic aggregates in response to CQ, DR5 seems not to be the sole integral membrane receptor trapped in lysosomal compartments upon this particular treatment (Supplementary Figures S3A, S3B and S4). Notably, although transferrin was found in lysosomes, no apparent co-localization with LC3 could be detected. In addition, intracellular staining of DR4 could only be accomplished using high concentrations of CQ (100 μM). Thus, the disruption of lysosomal function by agents such as CQ or Baf A delocalizes receptors that are normally destined for migration to the plasma membrane. Notably, using our experimental settings, Baf A is superior to CQ with respect to the capability of lysosomal de-acidification, in the presence and absence of 5-FU (Supplementary Figure S5A–S5D). In contrast to Baf A, CQ reduced lysosomal acidity only at early hours and in fact, after 24 h levels increased above controls. Possibly this is a result of organelle expansion. This indicates that the drug induced abrogation of lysosomal acidification may not be the main cause of receptor aggregation and inhibition of 5-FU toxicity.

### Divergent outcomes between 5-FU- and TRAIL-induced apoptosis in CQ pre-treated HCT116 cells indicate two separate mechanisms for DR5 activation

**Figure 5:**
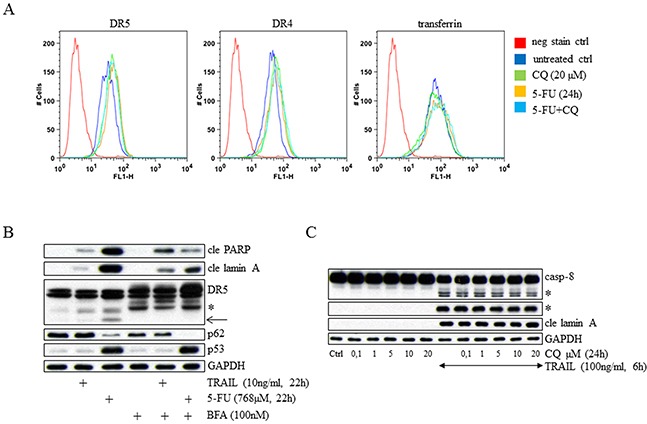
CQ cell treatment interferes with 5-FU- but not TRAIL-induced apoptosis Plasma membrane receptor levels were analyzed in FACS using live HCT116 *wt* cells, which were either left untreated or treated for 24h with CQ (20 μM) or 5-FU (768 μM), or their combination. Before FACS examination, 7-AAD (1 μg/mL) was added to the samples. Gating for 7-AAD negative cells allowed for immuno-detection of DR4, DR5 and transferrin exclusively in live cells **A.** Since all data in the figure are derived from the same experiment, the negative stain control (neg stain ctrl) have been used repeatedly. In **B.** the effect of brefeldin A (BFA; 100 nM) pre-treatment on TRAIL- or 5-FU-induced apoptosis was examined by SDS-PAGE using antibodies targeting DR5, cleaved PARP (cle PARP), cleaved lamin A (cle lamin A), p53 and p62. The consequences of CQ cell treatment (0.1 to 20 μM) for apoptosis stimulated by recombinant TRAIL were investigated by western blot and analysis of caspase-8 processing and cleaved lamin A. TRAIL (100 ng/mL) was added to cell cultures 6 h in advance of sample harvest **C.** Immuno-detection of GAPDH was used as a control for equal loading in (B and C). Processed caspase-8 fragments and the short isoform of DR5 are indicated by asterisks (B and C). An additional DR5-related fragment detected in western blot is indicated by an arrow (B).

An appealing explanation for the reduced 5-FU cytotoxicity detected in CQ-treated cells would be depletion of plasma membrane-located DR5 occurring as a result of lysosomal receptor accumulation. However, quantification of DR4, DR5 and transferrin in plasma membranes of living cells by flow cytometry analyzes revealed no gross reductions. Instead, increased amounts of DRs were revealed in response to CQ, 5-FU and their combination (Figure 5A). These data indicate that turnovers of the receptors analyzed are not sufficiently rapid to reduce their amounts in plasma membranes by CQ-induced cytoplasmic buildup. Secondly, unrestrained accumulation of receptors on the outermost cell membrane suggests that the source for lysosomal receptor aggregation is indeed outgoing endoplasmic reticulum (ER) trafficking. To further validate this assumption, abolition of the ER to Golgi protein transport was accomplished by pre-treating HCT116 cells with brefeldin A (BFA) before the addition of either 5-FU or the cognate DR5 ligand TRAIL. Similar to CQ, BFA reduced 5-FU cytotoxicity substantially, as observed by the diminished appearance of cleaved PARP and cleaved lamin A in western blotting. In contrast, the TRAIL and BFA co-treatment enhanced the appearance of the same apoptotic markers (Figure 5B), as previously described [18]. This not only confirms that cytosolic DR5 transport is important for 5-FU- generated apoptosis, but also indicates a distinct DR activation mechanism. In support of this, CQ concentrations ranging from 0.1 to 20 μM did not compromise apoptosis stimulated by TRAIL (Figure 5C). Thus, similar to the situation where DR5 accumulates in lysosome structures as a result of organelle targeting, inhibition of cytoplasmic receptor transport between ER and Golgi abrogates 5-FU cytotoxicity. Since cells treated with TRAIL respond differently during these experimental conditions, our data indicate two discrete mechanisms for DR5 activation.

### Cholesterol is an important factor for 5-FU-induced DR5 activation

**Figure 6:**
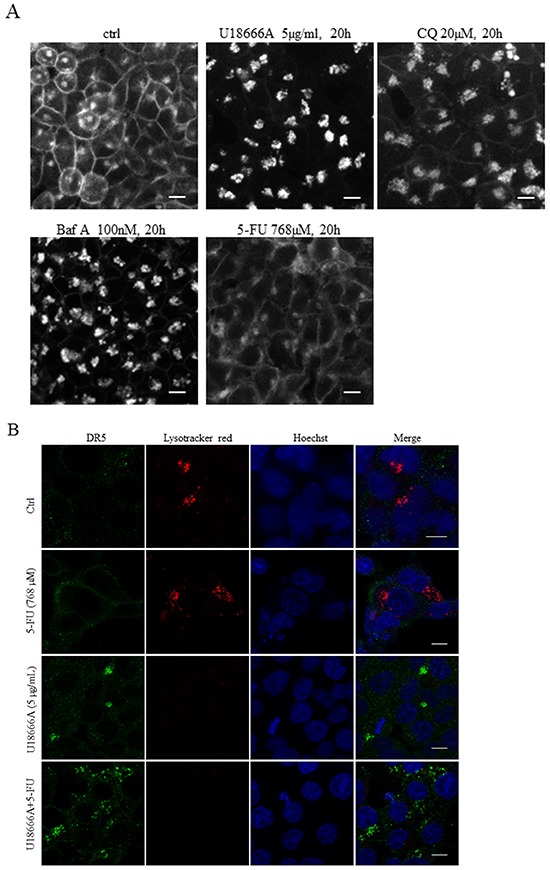

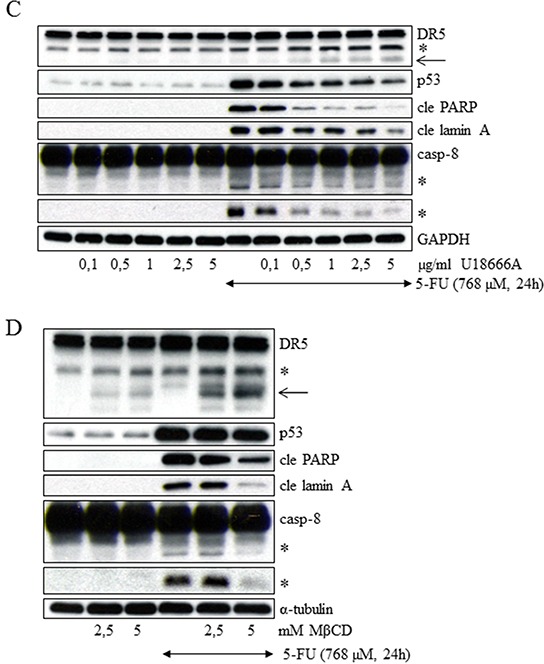
Free cholesterol is important for DR5 trafficking and 5-FU-induced apoptosis Filipin III, a fluorescent polyene macrolide antibiotic, was used to localize unesterified cholesterol in control, 5-FU (768 μM), Baf A (100 nM), CQ (20 μM) and U18666A (5 μg/mL)-treated and subsequently paraformaldehyde fixed HCT116 *wt* cells **A.** HCT116 control cells and cells induced with 5-FU, U18666A or their combination for 20 h were fixed in 3.8% formaldehyde for 20 min and exposed to a DR5 specific antibody (F2/B4, green). Before fixation, cells were incubated in the presence of lysotracker red (red, 100 nM) for 1 h. **B.** Cell nuclei were visualized by Hoechst 33342. Bars, 10 μm p53, cleaved PARP, lamin A (cle PARP, cle lamin A) and processing of caspase-8 were analyzed by immunoblotting of SDS-PAGE-separated cell lysates harvested 24 h post-treatment using either 5-FU (768 μM) alone, or in combination with U18666A (0.1-5 μg/mL) **C.** or MβCD (2.5 and 5 mM). **D.** GAPDH (C) and α-tubulin (D) served as a marker of equal sample loading. Processed caspase-8 fragments and the short isoform of DR5 are indicated by asterisks (C and D). An additional DR5-related fragment appearing in 5-FU and U18666A as well as 5-FU and MβCD co-treated cells is indicated by an arrow (C and D).

Two experimental approaches were taken in order to further elucidate the mechanism by which CQ restricts DR5 activity in 5-FU-generated apoptosis. Firstly, since acidic pH in the Golgi lumen is essential for correct protein glycosylation during their transit through the organelle and DR5 O-linked glycosylation controls tumor cell sensitivity to TRAIL [19, 20], benzyl-α-galNAc and PUGNAc were used to inhibit or enhance this post-translational modification, respectively. Keeping in mind that these agents not are directed to DR5 in particular, the western blot results clearly indicated that augmentation of O-glycosylation reinforced 5-FU-induced apoptosis; while limitation had the opposite effect (Supplementary Figures S6A and S6B). However, no alterations in DR5 localization similar to those provoked by disruption of lysosomal function were observed using benzyl-α-galNAc (Supplementary Figure S6C). Therefore, mechanisms other than aberrant DR5 O-linked glycosylation cause lysosomal accumulation. Secondly, apart from being an important factor for the integrity and fluidity of cell membranes, cholesterol also contributes to signal transduction and transport as a component of lipid rafts, caveolae and clathrin-coated pits [21]. The synthetic amphipathic steroid U18666A prevents intracellular cholesterol trafficking, resulting in lysosomal sterol accumulation (Figure 6A) [22]. Interestingly, filipin III, a fluorescent marker for unesterified cholesterol also indicated intracellular accumulation in CQ- and Baf A- but not 5-FU-treated cells (Figure 6A). U18666A used as a single agent generated intracellular DR5 aggregates in some cells. In combination with 5-FU, on the other hand, cytoplasmic receptor staining was more widespread (Figure 6B). Lysotracker red staining, a marker for organelle acidity, was undetectable in U18666A-treated cells using ICC (Figure 6B). However, in live cells analysis, lysosomal acidity remained, indicating the signal loss in fixed cells to be a methodological consequence (data not shown). Co-staining of DR5 and Lamp1 in ICC was therefore accomplished to verify lysosomal localization of receptor aggregates in U18666A-treated cells (Supplementary Figure S7). Furthermore, in support of the concept that DR5 transport involving membrane cholesterol is critical for 5-FU tumor cell toxicity, western blot detection of apoptotic markers clearly indicated U18666A as a potent inhibitor of the apoptotic process (Figure 6C). Methyl-β-cyclodextrin (MβCD), an agent that enhances cholesterol solubility and thereby cell depletion through the formation of inclusion complexes, verified the importance of cholesterol for the DR5 membrane traffic seemingly associated with 5-FU cytotoxicity (Figure 6D). Thus, cellular cholesterol traffic appears to be associated with DR5 transport and by this mean 5-FU-induced apoptosis.

## DISCUSSION

Autophagy has emerged as a critical process in the tumor cell response to chemotherapeutic drugs. The induction of autophagy in response to treatments may, however, provoke either cell death or survival. Thus, an anticancer effect or resistance may occur in a drug- or cell-specific manner, both being equally reliant on the recycling process [14]. For example, inhibition of autophagy sensitized non-small cell lung carcinoma cell lines to cisplatin-induced apoptosis although the response was attenuated in cells treated with etoposide [23]. A pro-death stimulus was also described in 5-FU-induced colon cancer cells, where inhibition of autophagy by Atg7 siRNA or 3-MA augmented apoptosis [24]. It has been suggested that secondary messenger molecules, including reactive oxygen species (ROS), cyclic AMP and inositol-3-phosphate are important for the crosstalk between autophagy and apoptosis [25]. Indeed, enhancement of cell death induced by chemotherapeutic agents under conditions of inhibited autophagy is, in some experimental conditions, ROS-dependent, as verified by the fact that antioxidants rescued cells from drug sensitization [15, 23]. In the current study, however, inhibition of autophagy *per se* does not influence apoptosis. An explanation for this inconsistency may be that extended 5-FU incubation is required in order for ROS to accumulate in consequence of failure to eliminate damaged mitochondria. Instead, we suggest that common autophagy inhibitory agents such CQ or Baf A force the accumulation of DR5 in lysosomal compartments which, in turn, reduces 5-FU-induced caspase-8 activity. TRAIL, on the other hand, remained a potent apoptosis inducer of CQ-treated cells, indicating that the source of lysosomal DR5 is the ER, a conclusion supported by the fact that inhibition of protein transport from the ER by BFA abrogates 5-FU cytotoxicity. The sorting of newly synthesized proteins in the trans-Golgi network via vesicles results in alternative transport destinations, including the extracellular space, different domains of the plasma membrane and lysosome-related organelles. The accumulation of DR5 in lysosomes occurring in the presence of CQ, Baf A or U18666A indicates an induction of abnormalities in the receptor transport mechanism. Alternatively, DR5 as well as DR4 and transferrin may encounter lysosomes for their natural turnover, or by means of lysosomal exocytosis, a Ca^2+^-regulated process required for the repair of plasma membrane disruptions [26]. Notably, we have previously established a role for calcium as a messenger for cell death signaling in response to 5-FU [27]. Thus, an explanation to the discrepancy between CQ and Baf A with respect to their potential to abrogate 5-FU-induced apoptosis might be provided by a differential inhibitory effect on lysosomal exocytosis. It is further important to emphasize that, based on ICC patterns of CQ-treated cells, DR5 seems to localize to lysosomal membranes rather than to the organelle interior as would be expected for proteins destined for destruction. Moreover, no overt enhancement of DR5 protein levels could be observed in cells treated with E64D or pepstatin A. The occurrence of cathepsins in the cytosol is sufficient to trigger apoptosis [28], and attenuation of lysosomal death signaling by organelle cholesterol accumulation generated by U18666A has been previously reported [29]. However, as 5-FU-induced apoptosis persisted in the presence of lysosomal protease inhibitors, release of lysosomal content could not serve as an explanation of our observations.

The use of U18666A has established an improved comprehension of intracellular cholesterol trafficking. Still, as this amphipathic steroid reduces cholesterol migration in between several cellular compartments [22], a more detailed interpretation of how DR5 and membrane cholesterol interact during treatment requires further investigations. The similarities between U18666A and CQ with respect to DR5 and 5-FU-toxicity must, however, be accentuated. It should also be noted that the range of CQ effects on cell physiology is not limited to lysosomal functions. For instance, CQ is considered to be a DNA intercalator and thereby able to protect cells from DNA-damaging chemotherapeutic agents such as doxorubicin and topoisomerase inhibitors [30]. 5-FU cytotoxicity, on the other hand, is a consequence of disturbances in nucleotide synthesis and using the present experimental model, we have previously described 5-FU-toxicity as RNA-related [13]. In summary, our data clearly indicate that a refined understanding of molecular crosstalk between lysosome-related processes and apoptotic cell death is essential in order to use the manipulation of autophagy as a tool in tumor chemotherapy.

## MATERIALS AND METHODS

### Cell culture

The HCT116 parental cell line and its p53-deficient variant (kindly provided by Professor Bert Vogelstein) were cultured in Dulbecco's modified Eagle's medium (DMEM) supplemented with 10% heat-inactivated FBS (Gibco) and PenStrep (100 U/ml penicillin, 100 μg/ml streptomycin; Sigma-Aldrich). Cell treatments with the antimetabolite 5-FU (Accord Healthcare Ltd), chloroquine (Sigma-Aldrich) or recombinant TRAIL (Thermo Fisher Scientific) were performed at concentrations and incubation times as indicated in the figures. In selected experiments the synthetic pan-caspase inhibitor N-benzyloxycarbonyl-Val-Ala-Asp(O-Me) fluoromethyl ketone (zVAD-fmk; 10 μM; Peptide Institute Inc.), E64d (10 μM), pepstatin A (7 μM), 3-methyladenine (3-MA; 5 mM), bafilomycin A (Baf A; 100 nM), U18666A (5 ug/mL), brefeldin A (BFA; 100 nM), PUGNAc (25-100 μM) or methyl-β-cyclodextrin (MCD; 5 mM) (all from Sigma-Aldrich) were added to cell cultures 1 h prior to drug treatment. Benzyl-α-galNAc (2.5 mM; Sigma Aldrich) was added to cultures 56 h in advance of 5-FU treatment.

### Gel electrophoresis and immunoblotting

Protein cell lysates were extracted by the cOmplete Lysis-M reagent (Roche) supplemented with protease (cOmplete ULTRA) and phosphatase (PhosSTOP) inhibitors (Roche). SDS-PAGE was performed according to previously described procedures [27].

### Expression vectors and retroviral transduction

The retroviral expression vectors pLXINhFADD and pXINhFLIP_L_ were described previously [31]. Retroviral particles were produced by transfection of the Phoenix-Ampho packaging cell line (kindly provided by Dr. G.P. Nolan, Stanford University, USA). Production of viral particles and retroviral transductions were performed as described [31]. Transduced cells were selected by treatment with 1 mg/ml of G418 (Life Technologies).

### Transmission electron microscopy (TEM)

Following fixation in 2.5% (w/v) glutaraldehyde in 0.1 M phosphate buffer (pH 7.4) for 30 min at RT and washing in 0.1 M phosphate buffer, cells were centrifuged and the pellets post- fixed in 2% (w/v) osmium tetroxide in 0.1 M phosphate buffer (pH 7.4) at 4°C for 2 h. Subsequent to dehydration in ethanol and then acetone, cells were embedded in LX-112 (Ladd). Ultrathin sections (~40–50 nm) were cut using a Leica EM UC 6 ultramicrotome. Sections were contrasted with uranyl acetate followed by lead citrate and examined in a Tecnai 12 Spirit Bio TWIN transmission electron microscope (FEI) at 100 kV. Digital images were created using a Veleta camera (Olympus Soft Imaging Solutions).

### Immunofluorescence

Immunocytochemistry was performed essentially according to previously described procedures [27]. As the target proteins exclusively were of transmembrane nature, the permeabilization step was omitted. Nuclear and plasma membrane co-stains were completed by incubating fixed cells in the presence of Hoechst 33342 (2 μg/mL, Sigma-Aldrich) and FITC-conjugated cholera toxin B (CTB; 0,2 μg/mL; Sigma-Aldrich), respectively, for 15 min in the dark. Similarly, free cholesterol was detected by Filipin III (50 μg/mL; Sigma Aldrich) in PBS supplemented with 10% FBS, incubating for 2 h in the dark. Lysosomes were visualized by means of LysoTracker® Red DND-99 (Molecular Probes^™^) according to the recommendations of the manufacturer. Briefly, 100 nM of the dye was added to the media of control or treated cells 30 min in advance of washing and fixing procedures. All samples were examined under a Zeiss LSM 510 META confocal laser scanning microscope (Carl Zeiss MicroImaging).

### Antibodies

The following primary antibodies were used in western blotting: p53 mAb, SQSTM1 (p62) mAb, Bcl-X_L_ pAb (Santa Cruz Biotechnology), LC3 pAb (MBL), GAPDH pAb (Trevigen), cleaved caspase-3 pAb, cleaved PARP mAb (Asp214), cleaved lamin A mAb, Atg5 pAb, Atg7 pAb, beclin pAb (Cell Signaling), tubulin mAb, DR5 pAb (Sigma-Aldrich), cFLIP mAb (Alexis), PARP mAb, TRAIL mAb, Ku80 mAb (BD Biosciences) and caspase-8 mAb (kindly provided by Profs P.H. Krammer and I. Lavrik (German Cancer Research Center, Heidelberg, Germany). Analysis of DRs in immunofluorescence and flow cytometry was performed using Fas pAb (Santa Cruz Biotechnology), DR4 and DR5 (F2/B4) mAbs (kindly provided by Dr. L. Anděra, Czech Academy of Sciences, Prague, Czech Republic). Transferrin was detected by the clone MEM-75 mAb (Abcam) and Lamp1 by the clone D2D11 mAb. Fluorescent secondary antibodies directed against mouse and rabbit (Alexa Fluor 488 and Alexa Fluor 594) were purchased from Molecular Probes^™^.

### RNA interference

Transfection of small interfering RNA's targeting p62 (siSQSTM1, s16961), negative control (siNegative Control No1) (Life Technologies), beclin, Atg5, Atg7 and negative control (D-00120613) ONTARGET-plus SMARTpool siRNAs (Dharmacon) was performed using the Lipofectamine RNAiMAX transfection reagent (Life Technologies) according to the manufacturer's instructions.

### Quantification of plasma membrane receptors

The level of plasma membrane receptors was detected in cells after incubation with specific antibodies using flow cytometry. Briefly, after washing with cold PBS supplemented with 5% FBS, cells were incubated for 30 minutes at 4°C with primary antibodies (anti-DR4, -DR5, and -transferrin) diluted 1:100 in washing solution. Cells were then washed twice, and incubated (30 min, 4°C) with the secondary antibody (AlexaFluor-488-conjugated donkey anti-mouse-IgG). After washing twice, the cells were stained for 15 min at 4°C with 7-AAD (1 μg/ml, Molecular Probes^™^) and analyzed by flow cytometry (FACScan, Becton Dickinson). The 7-AAD negative cells were subjected to receptor analysis (Cell Quest software). Green fluorescence measurements indicating the amount of the receptor present at the cell surface versus cell counts are visualized in histograms and related to controls lacking the specific primary antibody.

### Sub-G1 analysis

Cells were fixed in 70 % ethanol for 1 h at 4°C. Repeated washes in cold PBS and RNase A treatment (100 μg/ml, Invitrogen) for 1 h at 37°C were followed by propidium iodide staining (50 μg/ml, Sigma-Aldrich). Analysis in the FL3 channel in DDM mode was performed using the BD Accuri C6 system in combination with BD CSampler software (Becton-Dickinson).

## SUPPLEMENTARY FIGURES


