# Supplementary material for: Aberrant DR5 transport through disruption of lysosomal function suggests a novel mechanism for receptor activation
Source: Oncotarget. 2016 Aug 5;7(36):58286–301. doi: 10.18632/oncotarget.11073 (PMC5295431; doi:10.18632/oncotarget.11073)
Supplement: Supplementary file 1 [file oncotarget-07-58286-s001.pdf]

Aberrant DR5 transport through disruption of lysosomal function suggests a novel mechanism for receptor activation

SUPPLEMENTARY FIGURES

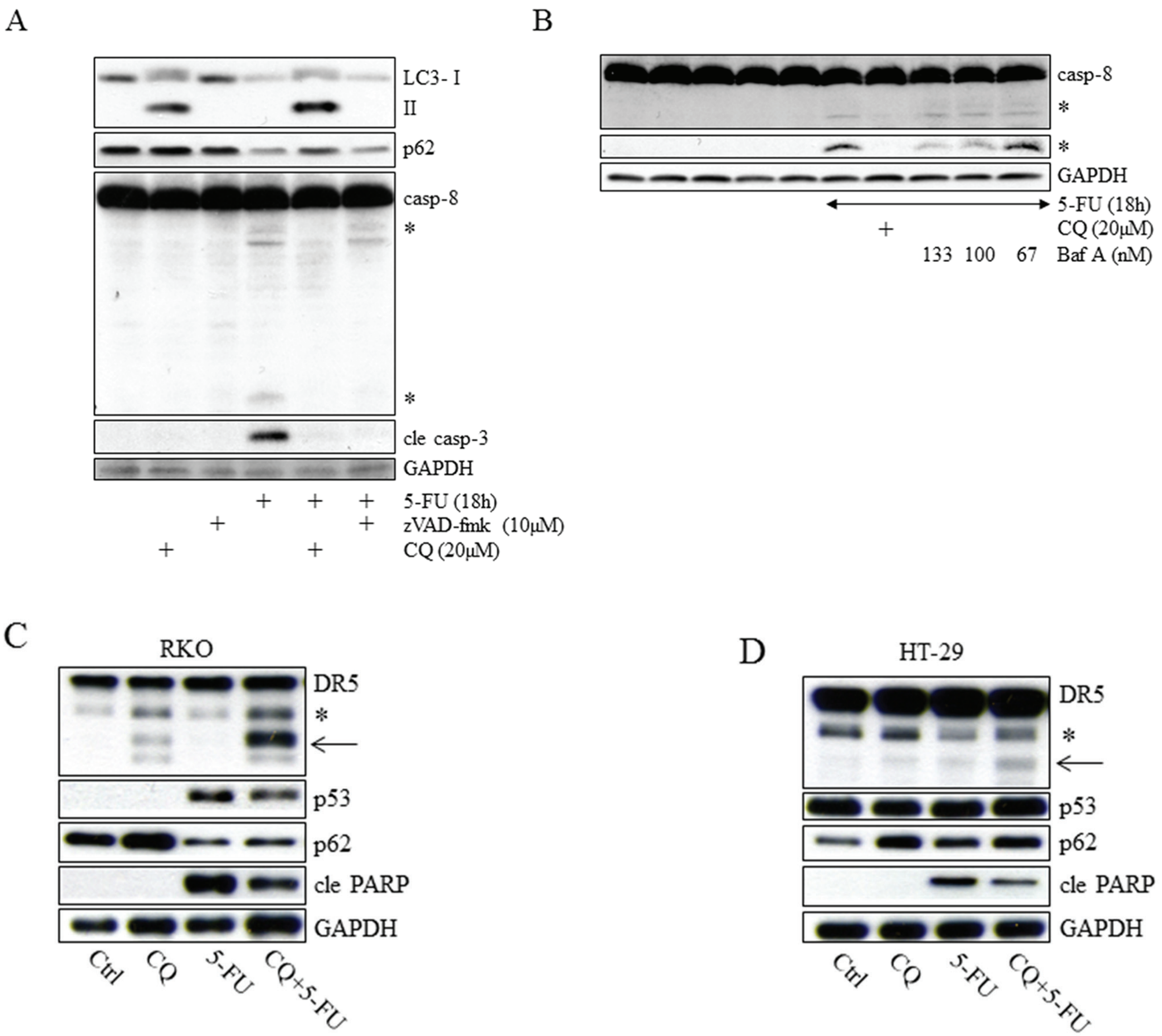

Supplementary Figure S1: Autophagy inhibition by chloroquine or bafilomycin A interferes with 5-FU cytotoxicity in HCT116, RKO and HT-29 cells. HCT116 wt cells were treated with 768 µM 5-FU for 18 h. In **A.** the effects of 20 µM chloroquine (CQ) or 10 µM zVAD-fmk with respect to the appearance of caspase-3 cleavage (cle casp-3) and caspase-8 processing were assessed by immunoblotting. In **B.** processing of caspase-8 generated by 5-FU alone or its combinations with CQ or Baf A (67-133 nM) was analyzed by western blotting. RKO **C.** and HT-29 **D.** cells were treated with 5-FU (768 µM, 26 h) and the effects of 10 µM CQ on the appearance of PARP cleavage (cle PARP), DR5, p53 and p62 were compared to relevant controls by immunoblotting of SDS-PAGE-separated protein lysates. GAPDH served as a marker for equal sample loading (A–D). Processed caspase-8 fragments and the short isoform of DR5 are indicated by asterisks (A–D). An uncharacterized DR5-related fragment appearing in CQ-treated samples is indicated by an arrow (C and D).

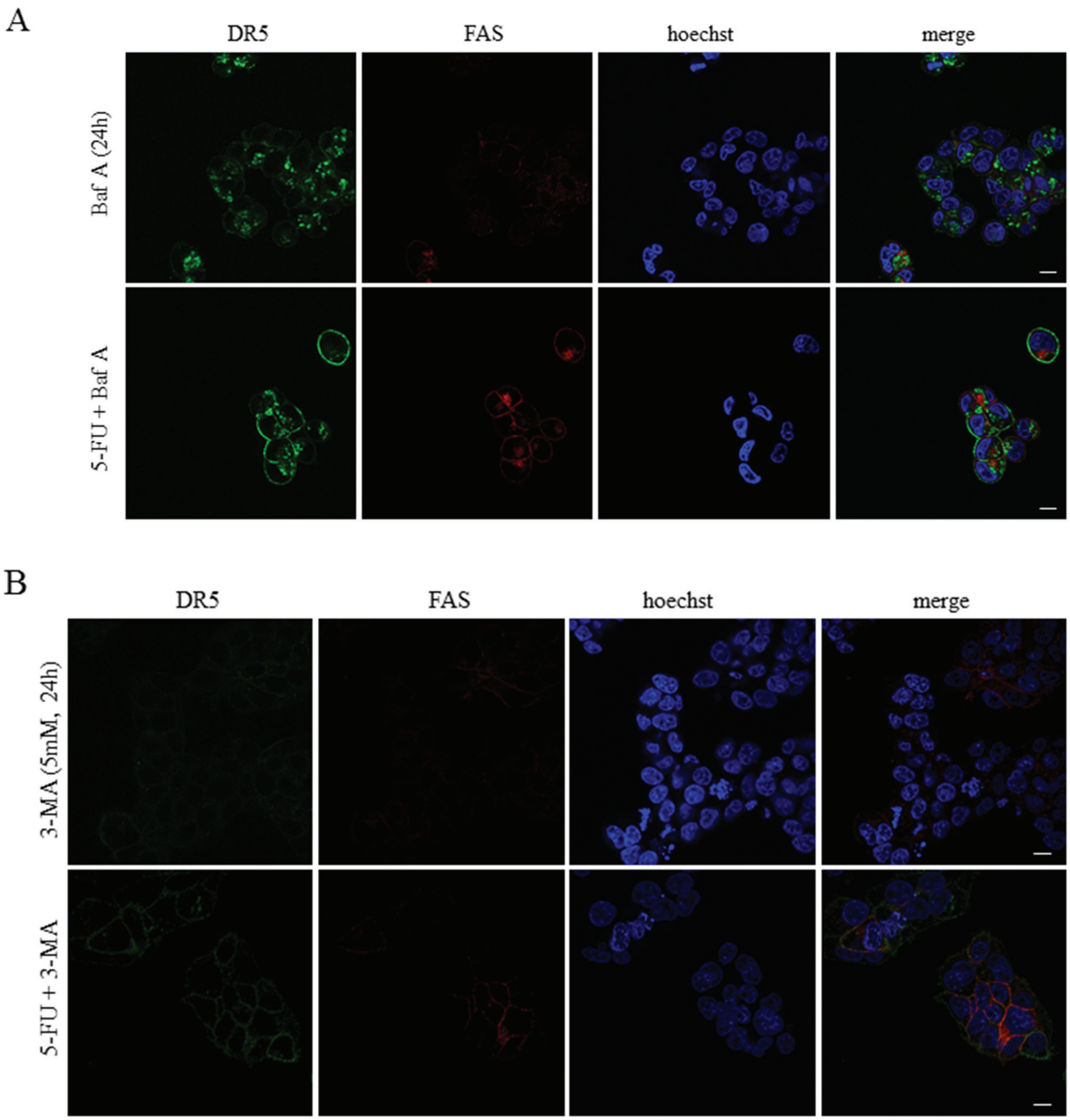

Supplementary Figure S2: Treatment with bafilomycin A but not 3-methyl adenine generates a punctuated DR5 cytoplasmic localization pattern which is distinct from FAS and analogous to the chloroquine cell response. HCT116 *wt* cells were treated with Baf A (100 nM) **A.** or 3-MA (5 mM) **B.** either alone or in combination with 5-FU (768 µM, 24 h). Following fixation in 3.8% formaldehyde immunostaining was performed using DR5 (green) and FAS (red) specific antibodies. Analysis was performed by confocal microscopy. Cell nuclei were counterstained by the Hoechst 33342 stain (blue). Bars, 10 µM.

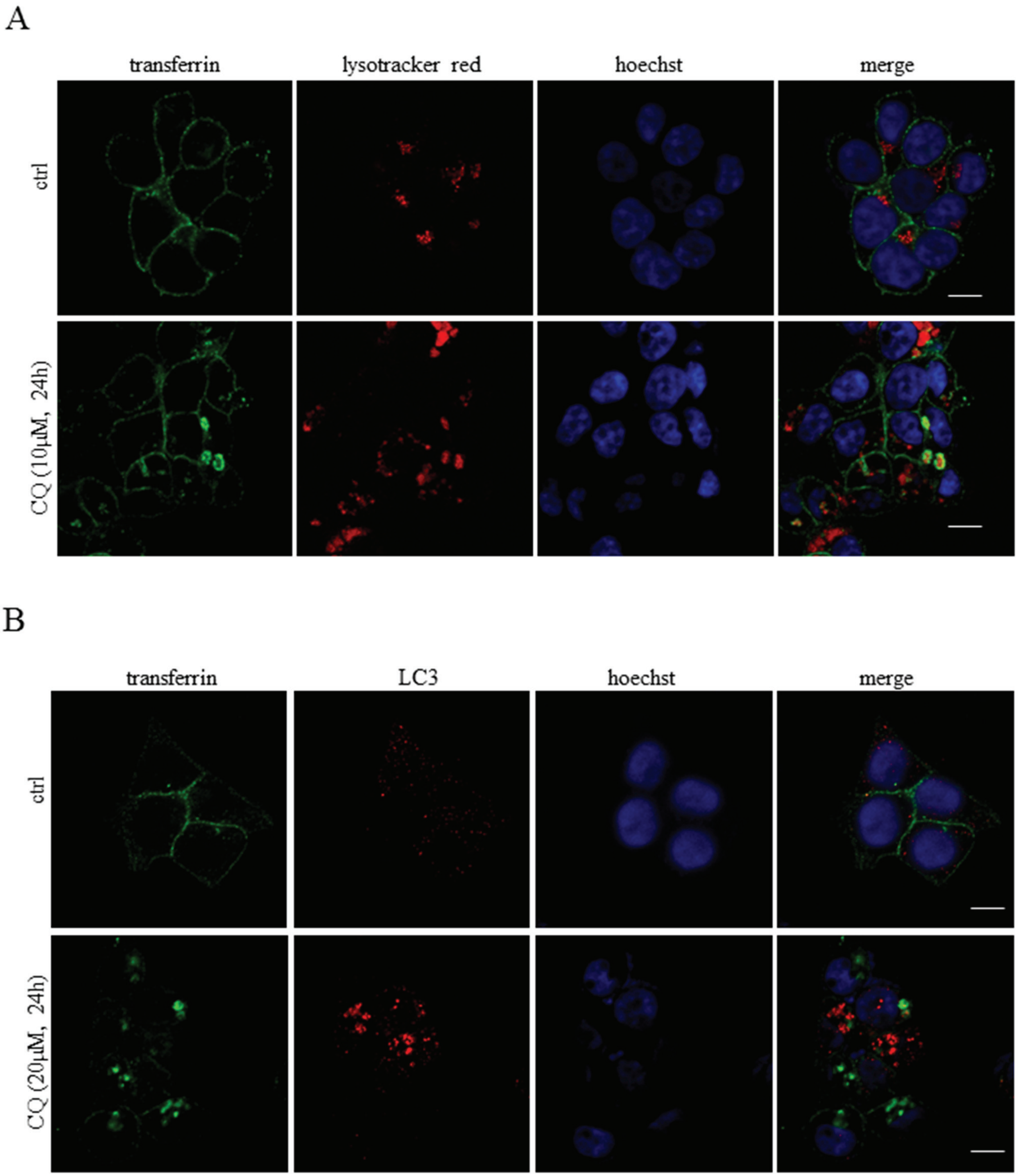

Supplementary Figure S3: Transferrin forms cytoplasmic aggregates in response to chloroquine, which co-localize to lysosomal but not autophagosomal compartments. HCT116 cells were either left untreated or treated with 10 μM CQ for 24 h. Subsequently, cells were fluorescently labeled and imaged using a confocal microscope. Green, transferrin; Blue, Hoechst-labeled nuclei. The relative localization of lysosomes and autophagosomes was investigated by counterstaining using Lysotracker **A.** and a LC3-specific antibody **B.** both visualized by red fluorescence. Bars, 10 μM.

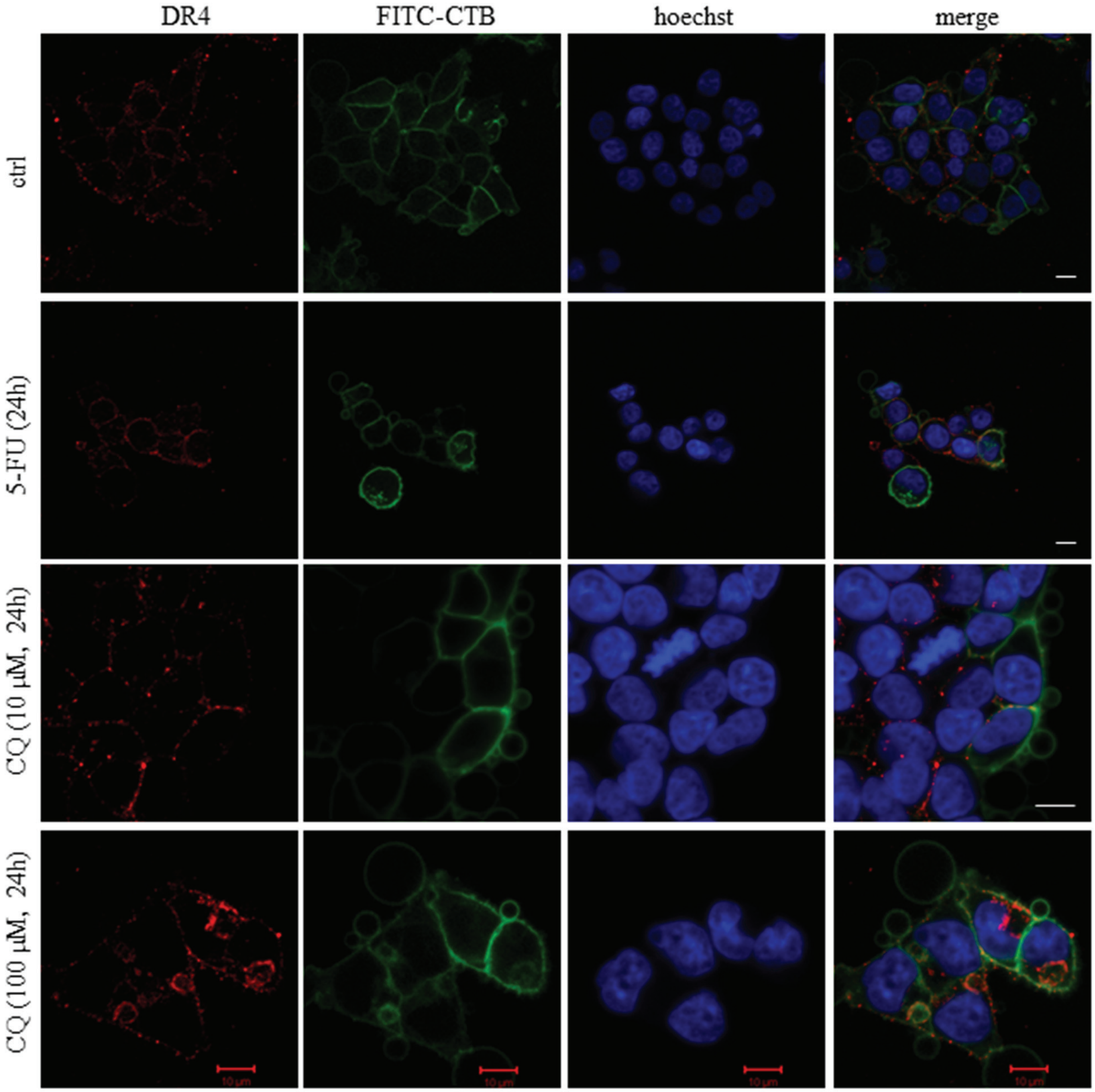

Supplementary Figure S4: Intracellular aggregates of DR4 are only formed at high chloroquine concentrations. Control HCT116 cells and cells treated with 10 or 100 µM CQ or 5-FU (768 µM) for 24 h were fixed in paraformaldehyde and then fluorescently labeled. Red, DR4; Green, FITC-cholera toxin B (CTB); Blue, Hoechst-labeled nuclei. CTB serves as marker for the plasma membrane. Bars, 10 µM.

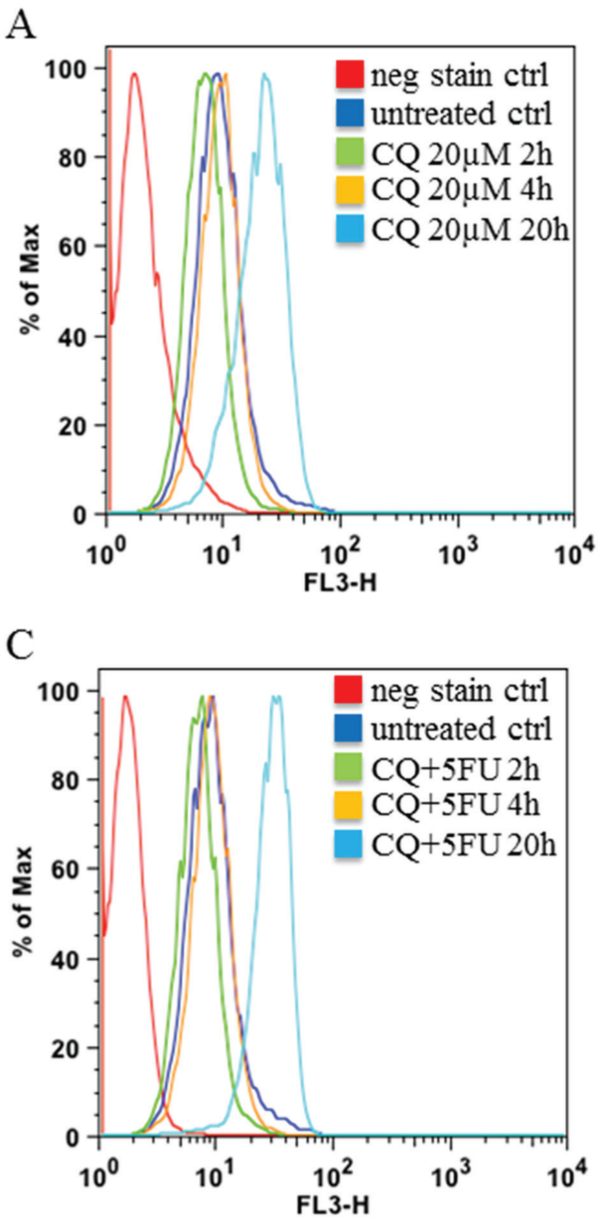

Supplementary Figure S5: Bafilomycin A is superior to chloroquine with respect to lysosomal de-acidification. HCT116 *wt* cells were treated as indicated in the figures **A–D.** Lysotracker red (100 nM) was added 1 h in advance of cell harvest. Fluorescence intensity was used as a measurement of lysosomal acidity. Analysis was performed on live cells by flow cytometry in the FL3-H channel. Since all data in the figure are derived from the same experiment, the negative stain control (neg stain ctrl) and the untreated control (untreated ctrl) have been used repeatedly.

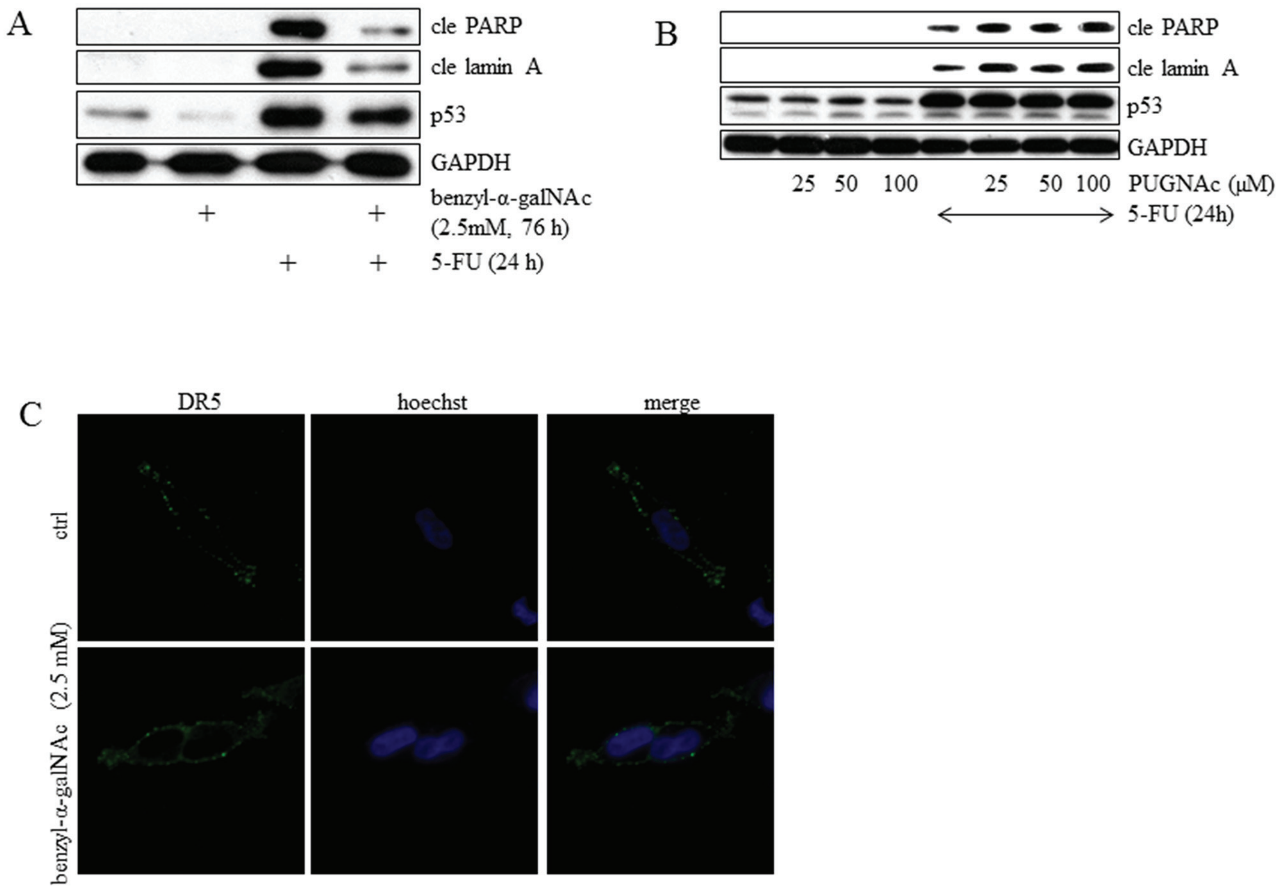

Supplementary Figure S6: Chemical manipulation of protein O-glycosylation affects 5-FU-induced apoptosis but does not alter DR5 localization. Along with controls HCT116 *wt* cells were treated with 5-FU (768 μM, 24 h) either as a single agent or in combination with 2.5 mM benzyl-α-galNAc **A,** or PUGNAc (25-100 μM) **B.** The effects of chemical inhibitors on the appearance of cleaved PARP (cle PARP) and cleaved lamin A (cle lamin A) as well as p53 were analyzed by western blotting. GAPDH served as a marker for equal sample loading. Control HCT116 cells and cells treated with 2.5 mM benzyl-α-galNAc for 76 h were fixed in paraformaldehyde and subsequently fluorescently labeled **C.** DR5, Green; Hoechst-labeled nuclei, Blue.

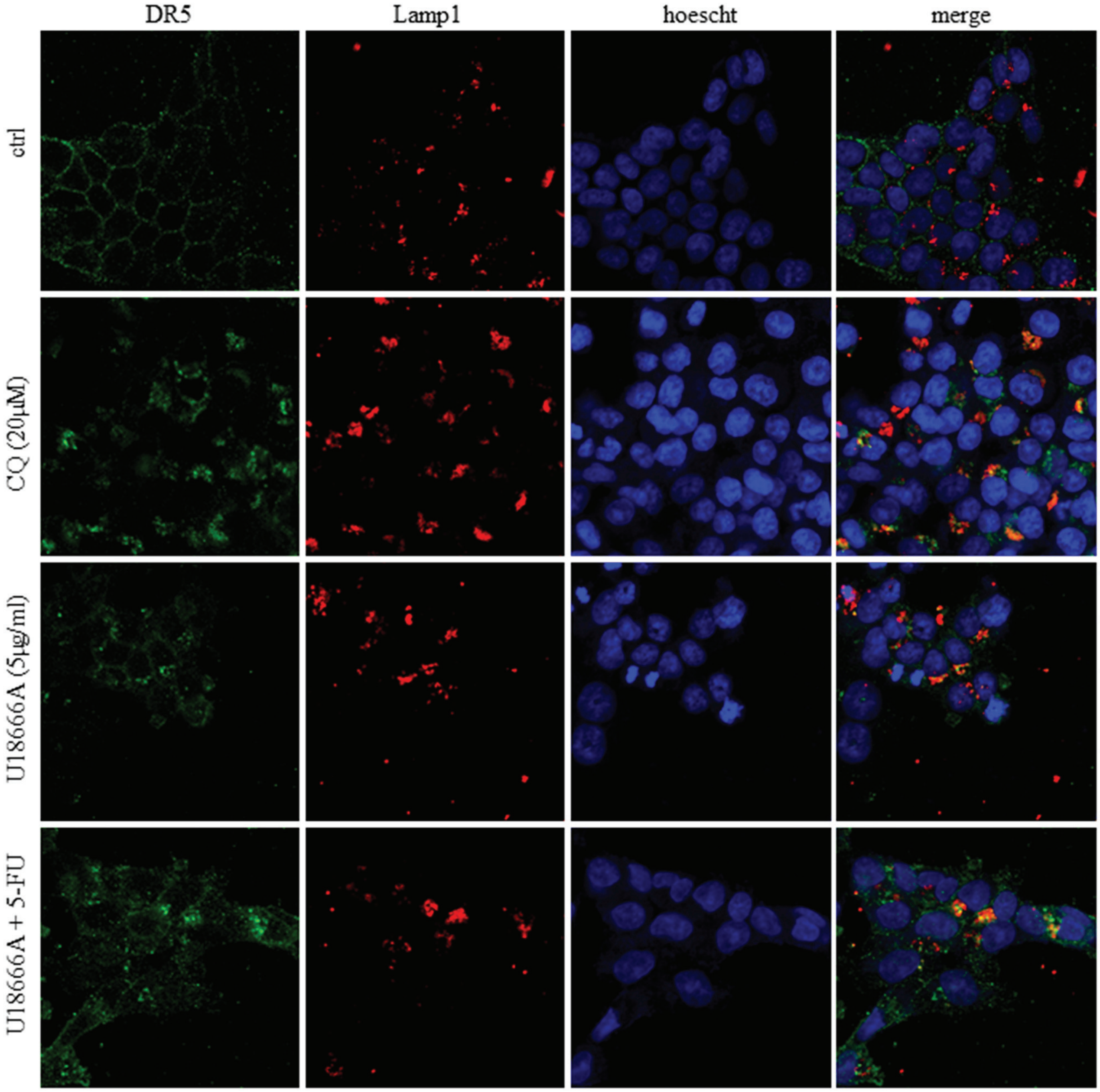

Supplementary Figure S7: U18666A treatment leads to cytosolic DR5 accumulations overlapping with lysosomes. Along with control cells, HCT116 *wt* cells treated with CQ (20 μM) or U18666A (5 μg/mL) alone or in combination with 5-FU (768 μM) for 24h. Fixation in 3.8 % formaldehyde was followed by staining of DR5 (green) and Lamp1 (red). All samples were analyzed by confocal microscopy. Cell nuclei were counterstained by the Hoechst 33342 stain (blue).
